# The Many Facets of Genetic Literacy: Assessing the Scalability of Multiple Measures for Broad Use in Survey Research

**DOI:** 10.1371/journal.pone.0141532

**Published:** 2015-10-28

**Authors:** Leah R. Abrams, Colleen M. McBride, Gillian W. Hooker, Joseph N. Cappella, Laura M. Koehly

**Affiliations:** 1 Social and Behavioral Research Branch, National Human Genome Research Institute, Bethesda, Maryland, United States of America; 2 Rollins School of Public Health, Emory University, Atlanta, Georgia, United States of America; 3 NextGxDx, Franklin, Tennessee, United States of America; 4 Annenberg School for Communication, University of Pennsylvania, Philadelphia, Pennsylvania, United States of America; Leibniz Institute for Prevention Research and Epidemiology (BIPS), GERMANY

## Abstract

**Objectives:**

To determine how three dimensions of genetic literacy (familiarity, skills, and factual knowledge) fit the hierarchy of knowledge outlined in E.M. Rogers’ *Diffusion of Innovations* to better conceptualize lay understandings of genomics.

**Methods:**

A consumer panel representing the US adult population (N = 1016) completed an electronic survey in November 2013. Adjusting for education, we used correlations, principle components analysis, Mokken Scale tests, and linear regressions to assess how scores on the three genetic literacy sub-dimensions fit an ordered scale.

**Results:**

The three scores significantly loaded onto one factor, even when adjusting for education. Analyses revealed moderate strength in scaling (0.416, p<0.001) and a difficulty ordering that matched Rogers’ hierarchy (knowledge more difficult than skills, followed by familiarity). Skills scores partially mediated the association between familiarity and knowledge with a significant indirect effect (0.241, p<0.001).

**Conclusion:**

We established an ordering in genetic literacy sub-dimensions such that familiarity with terminology precedes skills using information, which in turn precedes factual knowledge. This ordering is important to contextualizing previous findings, guiding measurement in future research, and identifying gaps in the understanding of genomics relevant to the demands of differing applications.

## Introduction

Applications of genomic discovery and technology are extending beyond the clinical setting into more and varied spaces. For example, there is increasing accessibility of genomic health information online [1, 2], and high profile celebrities are using entertainment media to share their medical choices based on personal genetic cancer risk [3, 4]. In addition to medical applications of genomics, the public encounters a host of consumer product advertising ranging from labels for genetically modified foods [5] to claims that skincare products can capitalize on genetic testing to more effectively reduce wrinkles [6]. Such exposures to genomic information outside of the clinical setting call upon the public to independently evaluate the veracity of these claims and make related decisions.

Consideration of the importance of “genetic literacy,” that is, sufficient knowledge and understanding of genetic principles to make decisions that sustain personal well-being and effective participation in social decisions on genetic issues [7], has increased. Yet the majority of these efforts have been focused on medical contexts. Several tools have been developed to measure genetic literacy, such as assessing pronunciation of genetic terminology [8] and technical knowledge about genes and heredity [9], but it remains unclear how these different measurements, all termed “genetic literacy,” relate to one another. In 2004, the Institute of Medicine published an extensive report on the broader concept of *health* literacy, highlighting its multidimensionality; the report called for better tools for measuring all aspects of health literacy to differentiate between lacking familiarity with language, lacking reading skills, and lacking substantive knowledge [10]. Such improvements in measurement are also relevant for genetic literacy and its broadening applications, and there is a growing demand for a new measurement approach that is not sample specific and can be administered through a survey [11].

The hierarchy of knowledge described in E.M. Roger’s seminal conceptualization of the diffusion of innovation offers a framework for considering how the public encounters the expanding applications of genomics in everyday scenarios beyond medical settings. Therefore, the levels of knowledge outlined by Rogers describing the diffusion of an innovation can be applied to the multiple dimensions of genetic literacy in the general population. Rogers holds that when a new innovation such as genomics is introduced, consumers experience stages of adoption in which they first ask “What is it?” (awareness knowledge), and “How does it work?” (how-to knowledge), followed by “Why does it work?” (principles knowledge) [12]. Diffusion theory suggests that answering one of these questions leads naturally to seeking the next level of knowledge. There is much to be gained from conceptualizing genetic literacy as a multidimensional knowledge scale like that proposed by Rogers. To start, establishing the scalability of sub-dimensions, or facets, of genetic literacy contextualizes disparate findings from previous research, allowing genetic literacy measurements from different tools or samples to be compared to each other. In addition, future research can use the probabilistic assumptions enabled by scaling to shorten survey length and strategically choose measurement tools that best fit the level of literacy required by a given application of genomic information. Finally, those interested in genomics education can use a leveled understanding of genetic literacy for baseline assessments, curriculum design that builds on existing knowledge, and learning evaluation.

Smerecnik and colleagues first established the utility of Rogers’ framework in the context of genomics through a literature review on public understandings of multifactorial genetic diseases [13], followed by an assessment of Rogers’ levels of knowledge in a Dutch consumer panel [14]. These findings empirically differentiated between sub-dimensions of genetic literacy and illustrated the usefulness of a knowledge hierarchy for highlighting incomplete understandings of genetic contributions to complex diseases. However, Smerecnik’s work is limited by having not assessed all three sub-dimensions as one scale and having focused exclusively on knowledge related to disease processes without due consideration of the broadening applications of genomic information. In addition, each measure in Smerecnik’s survey depended upon having completed the prior measure, which generates inherent dependencies between the dimensions of literacy and limits the ability of future researchers to independently assess a dimension relevant to a given context.

Our study aimed to build upon this work by applying Rogers’ hierarchy of knowledge to a broader conception of genetic literacy that includes applications in the market place and mass media, while using independently developed assessments of the levels of knowledge. In this context, awareness-knowledge is a general recognition of an innovation’s existence and is operationalized as familiarity with basic genomic terminology. How-to knowledge is one’s understanding of the way that an innovation functions, representing practical skills. How-to knowledge is measured as the ability to use basic genomic information like that which can be easily found online; this measure takes a similar approach to the widely used Newest Vital Sign, which assesses general health literacy as competency interpreting a provided nutrition label [15]. Principles-knowledge concerns the technicalities of why an innovation works and, in our study, comprises understanding of the technical facts about the biology of genes, heredity, and disease mechanisms. This type of knowledge has been captured by many of the existing genetic literacy assessment tools [7, 9, 16].

In this report, we asked the question: how can we characterize the state of genetic literacy in the American public in a way that captures the differing levels of knowledge relevant to the expanding applications of genomic technologies? We used a large consumer panel representative of the adult population in the U.S. to assess whether independent assessments of awareness, how-to, and principles knowledge tap into one common construct—genetic literacy. We then asked—does that construct capture more than general education level? And, can these three types of knowledge be scaled in accordance with Rogers’ suggested order? This rigorous examination of the multidimensionality of genetic literacy sets up for future work using this scale to understand how the public encounters genomics in increasingly diverse contexts.

## Methods and Materials

The Genetic Literacy Survey was designed to gather information about how the public understands genomics and applies this knowledge in non-technical settings. The research protocol and survey materials were deemed exempt by the NIH Office of Research Protections and at the University of Pennsylvania.

### Sample

Our sample (N = 1028) was drawn from a heterogeneous consumer panel compiled by GfK and designed to represent the adult population in the United States according to gender, age, education, and geographical region. The sample over-represents African Americans (26.8%) to ensure a large enough sub-sample for considering differences associated with race. We excluded respondents who refused all items within any key variable, resulting in an effective sample size of 1,016 respondents in this analysis.

### Survey

**Fig 1:**
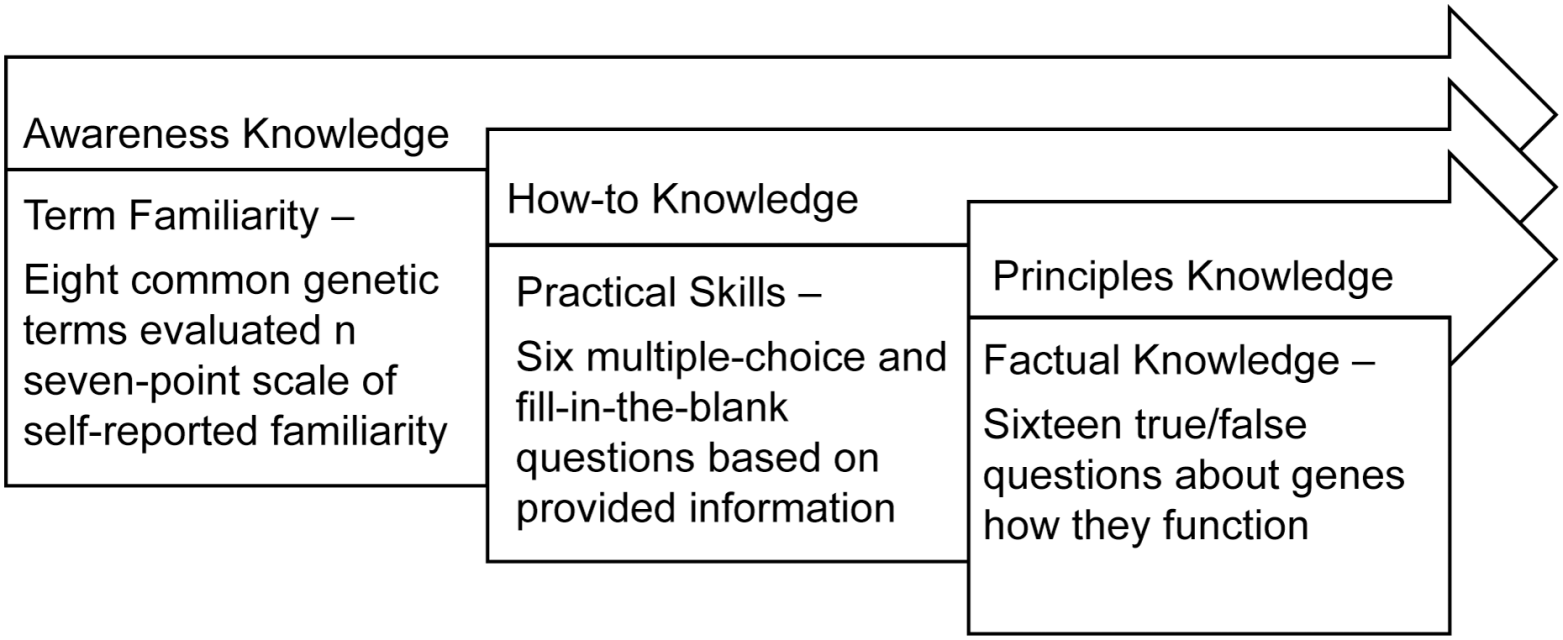
Applying Rogers’ levels of knowledge to Genetic Literacy Survey measures.

Respondents completed the electronic survey between November 5, 2013 and December 6, 2013. The survey was designed to take less than thirty minutes. The survey captured genetic literacy with three measures that each assess a different dimension of genomic knowledge to align with Rogers’ hierarchy (Fig 1).

#### “Awareness” assessed as term familiarity

The Genetic Literacy and Comprehension (GLAC) instrument presented eight terms commonly used when discussing genomics—genetic, chromosome, susceptibility, mutation, variation, abnormality, heredity, sporadic—and asked respondents to rate their general familiarity with each term from one (not at all familiar) to seven (completely familiar). These same eight terms are used in the short version of the Rapid Estimate of Adult Literacy in Genetics (REAL-G), which has established face and predictive validity [8]. While the REAL-G is aurally administered in a clinical settings to assess pronunciation, the GLAC tool was designed to be a quick and effective gauge of familiarity in written surveys [17]. In its original version, respondents also completed a fill-in-the blank question about each item; in this study, we used only the familiarity scores given that previous literacy research has shown the importance of subjective ability over more objective measures [18]. Scores were constructed based on respondents’ average perceived familiarity across the eight items (Range: 1 to 7), resulting in a Cronbach’s alpha of 0.951.

#### “How-to” assessed as practical skills

Respondents were presented with an information sheet that stated the purpose of *BRCA* genetic testing and the frequency of *BRCA* mutations in the population, along with a pictograph illustrating the relative breast cancer risk with and without this rare genetic mutation. This sheet was derived from the state of the science regarding optimal risk communication strategies [19–25]. Respondents could refer back to the provided information when answering six multiple choice and fill-in-the-blank questions, such as “What is the purpose of *BRCA* genetic testing?” and “About how many women out of 100 who do not have a *BRCA1* or *BRCA2* mutation will get breast cancer?” Scores were summed so that respondents received one point for each correct response; incorrect or unsure answers were coded as zero. Based on a Kuder-Richardson 20, scores demonstrated sufficient reliability with an alpha of 0.755.

#### “Principles” assessed as factual knowledge

The third measure presented sixteen technical statements about genes and how they function, seven of which were deliberately incorrect [7, 9, 16]. Respondents selected “Yes” if they thought the statement was true, “No” if they thought the statement was false, or “Do not know.” Prior research reported a reliability Cronbach’s alpha of 0.86 [9, 16]. In our study, respondents received one point for each correct response and no points for incorrect or unsure responses, demonstrating sufficient reliability with Kuder-Richardson 20 derived alpha of 0.842.

#### Education

Respondents were asked to report their highest grade level of education, which was categorized into 14 groups ranging from no formal education to professional or doctoral degree.

### Statistical Analysis

Analyses were performed using SPSS and SAS statistical software. To address our first two questions, we ran bivariate correlations and partial correlations adjusting for education. In addition, we conducted a Principle Components Analysis, first with the three scores, and then with the residual scores after removing the variance due to education.

To explore our third research question, we tested the hypothesis that the three genetic literacy measures form a scale in the order suggested by Rogers’ hierarchy of knowledge. We used Mokken scaling procedures [26] to assess whether high familiarity scores tend to precede high skills scores, which both precede high knowledge scores. Mokken scaling assumes dichotomous scores. In order to assess such scaling, we dichotomized each measure using cut points: 50%, 60%, 70% and 80% of total possible score. The results for Mokken scaling were consistent across these different cut-points, with the exception of the strict cut-off at 80%, due to limited variability in the high range of the factual knowledge scores. As such, we present those results associated with the 70% cut-point, a reasonably strict definition of “high literacy” that retains enough variability on each measure to detect scaling. Representing this 70% cut-point, “high familiarity” denotes mean responses of 5 or higher on the 7-point scale, “high skills” indicates five or six total correct responses, and “high knowledge” is considered twelve to sixteen correctly identified facts. To assess the strength and statistical significance of this scale, we computed a Loevinger H coefficient [27]; H coefficients between 0.3 and 0.4 indicate weak evidence of scaling; between 0.4 and 0.5 indicates moderate scaling; and greater than 0.5 provides strong evidence of scalability. Easiness scores, based on the percent achieving high scores on each measure, were used to rank the order of the dimensions of genetic literacy within the scale.

To test this same hypothesis with an alternate method, we fitted a series of regression models using the full quantitative range of scores on each measure while adjusting for education to determine if skills scores mediated the association between familiarity and knowledge. A Sobel test was conducted to estimate the indirect effect of familiarity on knowledge through the mediation of skills.

## Results

The characteristics of the sample (N = 1016) are described in Table 1. The range, mean, and percentage achieving high literacy for each measure are presented in Table 2, showing that respondents scored high on the familiarity measure more than on the skills measure, with the factual knowledge measure having the fewest high scores. On average, respondents were “somewhat familiar” with the genomic terms presented. Respondents were significantly more familiar with the terms “heredity” and “genetics” than all other terms (M = 5.4, SD = 1.87, p<0.001 and M = 5.52, SD = 1.77, p<0.001 respectively) and were significantly least familiar with the term “sporadic” (M = 4.17, SD = 2.35, p<0.001). The average score on the skills assessment was four of six correct responses, whereas on average, respondents correctly identified eight of sixteen facts.

**Table 1:** Sample Characteristics (N = 1016).

Characteristics	%(N)
**Gender**	
Male	47.7 (485)
**Race**	
White, non-His.	59.0 (599)
Black, non-His.	26.8 (272)
Hispanic	8.2 (83)
Other, non-His.	2.4 (24)
2+ races. non-His.	3.7 (38)
**Age**	
18–29	15.8 (161)
30–44	23.7 (241)
45–59	36.2 (368)
60+	24.2 (26)
**Education Level**	
Less than high school	9.0 (91)
High school	31.5 (320)
Some college	29.3 (298)
Bachelors or higher	30.2 (307)
**Income**	
Less than $24,999	18.7 (190)
$25,000–49,999	23.2 (236)
$50,000–99,999	31.9 (324)
$100,000+	26.2 (266)
**Region**	
Northeast	19.0 (193)
Midwest	22.9 (233)
South	37.7 (383)
West	20.4 (207)

**Table 2:** Descriptive statistics and Pearson’s Correlations of Genetic Literacy measures.

		Familiarity	Skills	Knowledge
**Descriptive Statistics**	**Range**	1–7	0–6	0–16
	**Mean (SD)**	4.98 (1.76)	3.94 (1.88)	8.39 (3.81)
	**% (N) scoring>70%**	57.6 (585)	47.5 (483)	22.2 (226)
**Pearson’s Correlations**	**Familiarity**	—	0.343, <0.001	0.451, <0.001
	**Skills**	0.405, <0.001	—	0.447, <0.001
	**Knowledge**	0.511, <0.001	0.507, <0.001	—
	**Education**	0.307, <0.001	0.308, <0.001	0.357, <0.001

Simple Pearson’s correlations shown below diagonal; partial correlations adjusting for education level shown above diagonal.

### Associations among literacy indicators and education

Each genetic literacy measure was significantly correlated with the others and with education (Table 2). When adjusting for education, the correlation among the three literacy measures remained significant with slight attenuation. The Principle Components Analysis produced similar results. All three measures loaded onto one factor, which explained 65% of the common variance between measures. Sixty-two percent of the variance in familiarity scores was extracted by this component, as were 62% of skills scores and 71% of factual knowledge scores. A second Principles Components Analysis was conducted with residual genetic literacy scores excluding the variance due to education; the three genetic literacy measures still loaded on one factor, which captured 61% of the total variance. In this second test, the majority of variance of each measure (58% of familiarity, 57% of skills, and 68% of factual knowledge) was extracted by this one common factor, representing an overarching domain of genetic literacy.

### Scalability of indicators of genetic literacy

**Fig 2:**
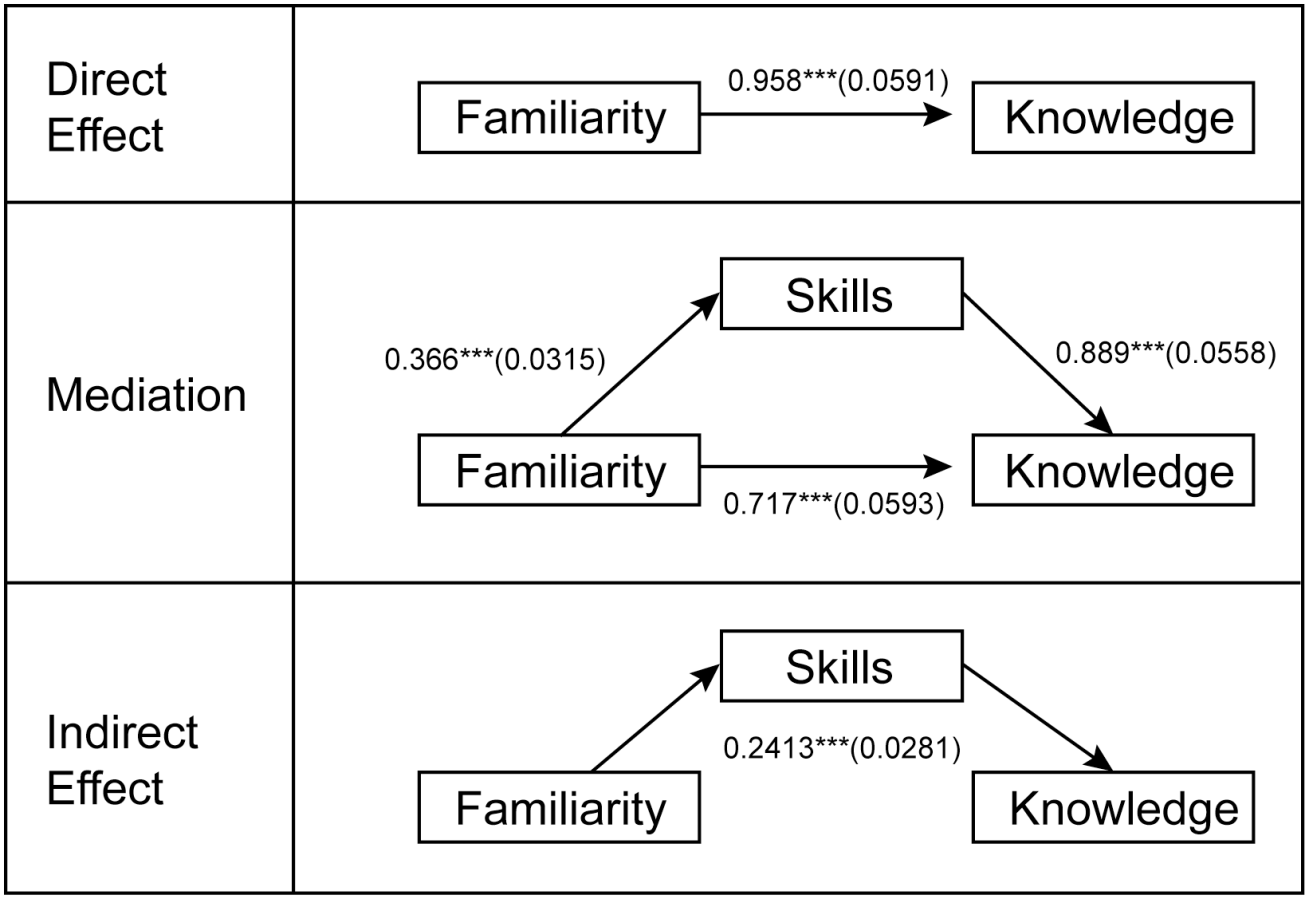
Mediation Model. Showing effect size (standard deviation); adjusting for education; * p<0.05. **p = 0.01, *** p<0.001.

Using Mokken Scaling, the Loevinger H Coefficient indicated the three genetic literacy measures to be significantly scalable with moderate strength (H = 0.416, p<0.001). In determining the fit of these sub-dimensions of genetic literacy to Rogers’ hierarchy of knowledge, the easiness coefficients support the hypothesized order—familiarity (0.58) has a higher easiness coefficient than skills (0.48), which both have higher easiness coefficients than factual knowledge (0.22). Using the full quantitative range of each measure, we tested whether skills mediates the association between familiarity and factual knowledge, adjusting for education level (Fig 2). We found evidence of partial mediation with a significant Sobel test for an indirect effect of familiarity through skills (0.241, p<0.001).

## Discussion

The first stage in deciding whether to adopt an innovation is to acquire knowledge—that is, being aware of the innovation and understanding how and why it works [12, 28]. This awareness and understanding of the innovation has been studied in the context of adopting new medical technologies, success in health promotion programs, and integration of technology into the classroom [29–31]. Genomics is one innovation that is rapidly moving beyond the confines of the clinic setting. As such, consumers increasingly encounter genomic information in every day contexts and must make decisions about whether and how they want to integrate this new technology into their lives. Genetic literacy reflects the knowledge necessary for making such decisions. Our study builds on previous applications of Rogers’ hierarchy of knowledge [14] by providing an overview of genetic literacy in the U.S. general public and exploring the scalability of independently developed genetic literacy indicators. Overall, we found that the three measures used in our survey tap into one main component, even without the contribution of education. Further, we found evidence that each sub-dimension of genetic literacy differs in “easiness” in the order put forward by Rogers, forming a moderately strong Mokken scale. These findings are in line with, and build upon, those of Smerencik and colleagues [14], with three main implications—contextualizing previous findings on genetic literacy, providing tools for future research, and enhancing genomics education.

To start, previous research has tended to measure only one of the sub-dimensions of genetic literacy as a proxy for the larger construct, resulting in over- or under-estimations that can now be contextualized within our multidimensional scale. For example, one study measured familiarity with genomic terminology (awareness knowledge) to examine its relation to behavioral motivation following genetic risk results; this study determined that 38% of the highly educated sample had inadequate genetic literacy based on familiarity scores [32]. However, according to Rogers, how-to knowledge plays the essential role in the trial and decision stage of adopting an innovation—here, the use of genetic test results to guide behavior [12]. Therefore, having practical skills to interpret provided risk information is likely a more salient dimension of genetic literacy for this scenario. Our scaling results suggest that the 62% of this sample with adequate awareness knowledge may not all have adequate how-to knowledge. Therefore, claims of low genetic literacy using familiarity indicators can be considered underestimations, having assessed awareness knowledge, which is the lowest level of knowledge in Roger’s knowledge hierarchy.

Similarly, findings that highlight a lack of factual knowledge about genomics should be considered with the understanding that those who do not know such technical facts may possess awareness and practical knowledge. Awareness and practical, or how-to, knowledge may serve well enough for certain contexts. For example, there is evidence that awareness and how-to knowledge are important to the adoption of health promoting behaviors and medical innovations [31]. Consumer applications of genomic information, for example, may not require the public to understand the science of how genes function. Rogers explains that it is often possible to adopt an innovation without understanding the principles that underlie it, though this high-level of knowledge can help facilitate proper and long-term use [12]. In the context of genomics, principles knowledge might be important for those affected by genetic disorders to understand their treatment and their family’s risk of inheritance. Principles knowledge is certainly essential for early adopters and change agents, such as clinicians and educators [28, 30]. However, for consumer applications of genomics, understanding the words used in an advertisement (awareness knowledge) and being able to find and use additional information online (how-to knowledge) might be a more important dimensions of genetic literacy than technical facts. In this sense, a multidimensional conception of genetic literacy counters the dated deficit model, which has been criticized in health literacy literature for overemphasizing basic scientific principles and underemphasizing that which the public already knows [33].

Clearly, our results have the potential to inform how genetic literacy is measured in future research. Unlike previous applications of Rogers’ knowledge hierarchy to genomics, our measures of each level of genetic literacy are independent from each other so that researchers can apply only the most relevant indicator for their given contexts. An important next step in this work is to determine thresholds on each sub-dimension of literacy that represent adequate levels of knowledge to effectively use varied applications of genomics—from confidently interpreting labels on genetically modified salmon to making health decisions using genetic risk feedback. For example, if a study wanted to predict the success of a marketing campaign for a wrinkle cream with a genetically tailored formula, it could ask—will consumers adopt this innovation based on familiarity with the genomic terminology used in the advertisements and understanding of how to use the cream? Or, do customers need to understand why genetic tailoring is effective to be convinced to buy the product?

In addition, our Mokken scale results allow for survey length in future research to be reduced because of the probabilistic inference that those achieving high scores on a given measure likely possess the preceding levels of knowledge [34]. For example, if a pre-semester assessment seeks to evaluate principles knowledge regarding genomics in an undergraduate college class to inform curriculum development, those students with high scores can be assumed to also have high awareness knowledge and high how-to knowledge. In that sense, establishing scalability enables researchers to infer more from these results without added questions. However, the probabilistic nature of Mokken scaling prevents the presented order from being interpreted as definite.

A third set of implications from our results relate to improving genomics education. Our scale can be used to inform the stages of efforts that gauge understandings of genomics and build up stepwise through the sequential dimensions of genetic literacy. Such applications for assessing gaps or flaws in knowledge and evaluating learning outcomes in genetic counseling or public health programming are obvious. Outside of the medical context, this genetic literacy scale could enable a court system to assess jury members’ genetic literacy level and then efficiently bring them to a sufficient knowledge level without wasting resources re-teaching existing knowledge or teaching irrelevant dimensions literacy.

One limitation of our study is the piloted use of the practical skills measure, though we found strong reliability among the six items. We chose to assess the ability to interpret genomic information, which has previously been a gap in the literature, because of how technology is increasing users’ control in seeking and processing risk information [35], as seen in the proliferation of genomic health information online [2, 36]. The information sheet presented in our skills measure uses the latest findings in optimal risk communication strategies [19–25] to capture the use of information like one might encounter in a doctor’s office or on a government webpage. However, respondents could reference the provided information sheet as much or as little as they liked, and thus, skills scores could partially reflect compliance and carefulness rather than ability. Our research contextualizes this important new measurement by comparing it to other types of genetic literacy that have been measured in the past.

Our research has strength in its theoretical grounding and methodological rigor, as it establishes the relations of all three levels of knowledge rather than comparing or distinguishing between two at a time. This research responds to recent calls for a systematic national assessment of genetic literacy [11] by using a representative sample of the U.S. adult population to provide baseline data that future researchers can reference. Future research can consider how these results compare to the state of genetic literacy worldwide. The multidimensional conceptualization of genetic literacy put forth in this research can be applied in the future studies that assess how the public uses genomic information in an expanding range of settings.

## Supporting Information

S1 Dataset Genetic Literacy Survey Dataset. (CSV)

S1 File Codebook for the Genetic Literacy Survey Dataset. (PDF)
