# Supplementary material for: The Many Facets of Genetic Literacy: Assessing the Scalability of Multiple Measures for Broad Use in Survey Research
Source: PLoS One. 2015 Oct 28;10(10):e0141532. doi: 10.1371/journal.pone.0141532 (PMC4625002; doi:10.1371/journal.pone.0141532)
Supplement: S1 File — (PDF) [file pone.0141532.s002.pdf]

Genetic Literacy Public Survey: 2013 [United States]

The purpose of the study is to measure three facets of genetic literacy, term familiarity, practical skills, and factual knowledge, in a probability sample of U.S. adults; the sample over-represents African Americans. The aim is to determine whether these facets fit the hierarchy of knowledge outlined within E.M. Rogers' *Diffusion of Innovations* and to characterize lay understandings of genomics. The study is funded by the National Institutes of Health' National Human Genome Research Institute (NHGRI).

The data are provided in the file: GeneticLiteracySurvey.csv; these files includes variables names in the first row.

The dataset includes 1016 respondents who provided measurements on the three genetic literacy scales between November 5, 2013 and December 6, 2013.

Scale scores were computed in the following way:

Term Familiarity— The Genetic Literacy and Comprehension (GLAC) instrument presented eight terms commonly used when discussing genomics—genetic, chromosome, susceptibility, mutation, variation, abnormality, heredity, sporadic— and asked respondents to rate their general familiarity with each term from one (not at all familiar) to seven (completely familiar). These same eight terms are used in the short version of the Rapid Estimate of Adult Literacy in Genetics (REAL-G), which has established face and predictive validity (Erby, Roter, Larson, & Cho, 2008). While the REAL-G is aurally administered in a clinical settings to assess pronunciation, the GLAC tool was designed to be a quick and effective gauge of familiarity in written surveys (Hooker et al., 2014). In its original version, respondents also completed a fill-in-the blank question about each item; in this study, we used only the familiarity scores given that previous literacy research has shown the importance of subjective ability over more objective measures (Miron-Shatz, Hanoch, Doniger, Omer, & Ozanne, 2014). Scores were constructed based on respondents' average perceived familiarity across the eight items (Range: 1 to 7), resulting in a Cronbach's alpha of 0.951.

High/low Familiarity—low (coded as 1) is below 70% cut off on familiarity scale (1-4), whereas high (coded as 2) is above (5-7).

Practical Skills— Respondents were presented with an information sheet that stated the purpose of *BRCA* genetic testing and the frequency of *BRCA* mutations in the population, along with a pictograph illustrating the relative breast cancer risk with and without this rare genetic mutation. This sheet was derived from the state of the science regarding optimal risk communication strategies (Garcia-Retamero, Okan, & Cokely, 2012; Hawley et al., 2008; Price, Cameron, & Butow, 2007; Schwartz et al., 2009; Tait, Voepel-Lewis, Zikmund-Fisher, & Fagerlin, 2010; Zikmund-Fisher, Fagerlin, & Ubel, 2008; B. J. Zikmund-Fisher et al., 2008). Respondents could refer back to the provided information when answering six multiple choice and fill-in-the-blank questions:

1. What is the purpose of *BRCA* genetic testing?
2. About how many women out of 100 who do not have a *BRCA1* or *BRCA2* mutation will get breast cancer?
3. Please select the phrase that best completes the following statement: Harmful mutations in the *BRCA1* and *BRCA2* genes are…

4. About what percentage of women who have a *BRCA1* or *BRCA2* mutation will get breast cancer?
5. Who is less likely to get breast cancer?
6. Which of the following groups is a larger part of the general population?

Scores were summed so that respondents received one point for each correct response; incorrect or unsure answers were coded as zero (Range: 0 to 6). Based on a Kuder-Richardson 20, scores demonstrated sufficient reliability with an alpha of 0.755.

High/low Skills—low (coded as 1) is below 70% cut off (0-4), high (coded as 2) is above this cut off (5-6).

Genetics Knowledge—Participants were presented with sixteen technical statements about genes and how they function, seven of which were deliberately incorrect (Bowling et al., 2008; Haga et al., 2013; Jallinoja & Aro, 1999). Respondents selected "Yes" if they thought the statement was true, "No" if they thought the statement was false, or "Do not know." Prior research reported a reliability Cronbach's alpha of 0.86 (Haga et al., 2013; Jallinoja & Aro, 1999). In our study, respondents received one point for each correct response and no points for incorrect or unsure responses, demonstrating sufficient reliability with Kuder-Richardson 20 derived alpha of 0.842. Scores ranged from 0 to 16.

High/low knowledge—low (coded as 1) was below 70% cut off (0-11), high (coded as 2) was above cut off (12-16).

Education - Respondents were asked to report their highest grade level of education, which was categorized into 14 groups ranging from no formal education to professional or doctoral degree. These 14 groups were further categorized into 4 categories: 1) Less Than High School Education; 2) High School Graduate; 3) Some College Education; 4) Bachelor's Degree or Higher.

Demographic Characteristics – Those included in the dataset are age, gender, race/ethnicity, household income, and geographic region of residence in the United States.

The following variables are provided in the csv file:

TYPE OF VARIABLE	NAME	NAME IN DATASET	VALUES
Facets of Genetic Literacy	Term Familiarity	Term_Familiarity	Average Term Familiarity Score; Range: 1 to 7
	High/Low Familiarity	High_Familiarity	1 = Low Familiarity<br>2 = High Familiarity
	Practical Skills	Practical_Skills	Number of items correct; Range: 1 to 6
	High/Low Skills	High_Skills	1 = Low Practical Skills<br>2 = High Practical Skills
	Genetics Knowledge	Genetics_Knowledge	Number of items correct; Range: 1 to 16
	High/Low Knowledge	High_Knowledge	1 = Low Knowledge<br>2 = High Knowledge
	Highest Educational Degree	PPEDUC	1 = No Formal Education<br>2 = 1st, 2nd, 3rd, or 4th grade<br>3 = 5th or 6th grade<br>4 = 7th or 8th grade<br>5 = 9th grade<br>6 = 10th grade<br>7 = 11th grade<br>8 = 12th grade<br>9 = High school diploma or equivalent<br>10 = Some college, no degree<br>11 = Associate degree<br>12 = Bachelor’s degree<br>13 = Master’s degree<br>14 = Professional or Doctorate degree

Sample Characteristics	Education - Categorized	PPEDUCAT	1 = Less than high school<br>2 = High school<br>3 = Some college<br>4 = Bachelor's degree or higher
	Race	PPETHM	1 = White, Non-Hispanic<br>2 = Black, Non-Hispanic<br>3 = Other, Non-Hispanic<br>4 = Hispanic<br>5 = 2+ Races, Non-Hispanic
	Chronological Age	PPAGE	Chronological Age, in years
	Age - Categorized	ppagect4	1 = 18 to 29 years<br>2 = 30 to 44 years<br>3 = 45 to 59 years<br>4 = 60+ years<br>99 = Under 18 years
	Gender	PPGENDER	1 = Male<br>2 = Female
	Income	PPINCIMP	1 = Less than $5,000<br>2 = $5,000 to $7,499<br>3 = $7,500 to $9,999<br>4 = $10,000 to $12,499<br>5 = $12,500 to $14,999<br>6 = $15,000 to $19,999<br>7 = $20,000 to $24,999<br>8 = $25,000 to $29,999<br>9 = $30,000 to $34,999<br>10 = $35,000 to $39,999<br>11 = $40,000 to $49,999<br>12 = $50,000 to $59,999<br>13 = $60,000 to $74,999<br>14 = $75,000 to $84,999<br>15 = $85,000 to $99,999<br>16 = $100,000 to $124,999<br>17 = $125,000 to $149,999<br>18 = $150,000 to $174,999<br>19 = $175,000 or more

	Region	PPREG4	1 = Northeast<br>2 = Midwest<br>3 = South<br>4 = West

References

Bowling, B. V., Acra, E. E., Wang, L., Myers, M. F., Dean, G. E., Markle, G. C., . . . Huether, C. A. (2008). Development and evaluation of a genetics literacy assessment instrument for undergraduates. *Genetics, 178*(1), 15-22. doi: 10.1534/genetics.107.079533

Erby, L. H., Roter, D., Larson, S., & Cho, J. (2008). The rapid estimate of adult literacy in genetics (REAL-G): a means to assess literacy deficits in the context of genetics. *Am J Med Genet A, 146a*(2), 174-181. doi: 10.1002/ajmg.a.32068

Garcia-Retamero, R., Okan, Y., & Cokely, E. T. (2012). Using visual aids to improve communication of risks about health: a review. *The Scientific World Journal, 2012*.

Haga, S. B., Barry, W. T., Mills, R., Ginsburg, G. S., Svetkey, L., Sullivan, J., & Willard, H. F. (2013). Public knowledge of and attitudes toward genetics and genetic testing. *Genet Test Mol Biomarkers, 17*(4), 327-335. doi: 10.1089/gtmb.2012.0350

Hawley, S. T., Zikmund-Fisher, B., Ubel, P., Jancovic, A., Lucas, T., & Fagerlin, A. (2008). The impact of the format of graphical presentation on health-related knowledge and treatment choices. *Patient Educ Couns, 73*(3), 448-455. doi: http://dx.doi.org/10.1016/j.pec.2008.07.023

Hooker, G. W., Peay, H., Erby, L., Bayless, T., Biesecker, B. B., & Roter, D. L. (2014). Genetic literacy and patient perceptions of IBD testing utility and disease control: a randomized vignette study of genetic testing. *Inflamm Bowel Dis, 20*(5), 901-908. doi: 10.1097/mib.0000000000000021

Jallinoja, P., & Aro, A. R. (1999). Knowledge about genes and heredity among Finns. *New Genetics and Society, 18*(1), 101-110.

Miron-Shatz, T., Hanoch, Y., Doniger, G. M., Omer, Z. B., & Ozanne, E. M. (2014). Subjective but not objective numeracy influences willingness to pay for BRCA1/2 genetic testing. *Judgment and Decision Making, 9*(2), 152-158.

Price, M., Cameron, R., & Butow, P. (2007). Communicating risk information: The influence of graphical display format on quantitative information perception—Accuracy, comprehension and preferences. *Patient Education and Counseling, 69*(1–3), 121-128. doi: http://dx.doi.org/10.1016/j.pec.2007.08.006

Schwartz, M. D., Valdimarsdottir, H. B., DeMarco, T. A., Peshkin, B. N., Lawrence, W., Rispoli, J., . . . Komaridis, K. (2009). Randomized trial of a decision aid for BRCA1/BRCA2 mutation carriers: Impact on measures of decision making and satisfaction. *Health Psychology, 28*(1), 11-19. doi: 10.1037/a0013147

Tait, A. R., Voepel-Lewis, T., Zikmund-Fisher, B. J., & Fagerlin, A. (2010). The Effect of Format on Parents' Understanding of the Risks and Benefits of Clinical Research: A Comparison between Text, Tables, and Graphics. *J Health Commun, 15*(5), 487-501. doi: 10.1080/10810730.2010.492560

Zikmund-Fisher, B. J., Fagerlin, A., & Ubel, P. A. (2008). Improving understanding of adjuvant therapy options by using simpler risk graphics. *Cancer, 113*(12), 3382-3390. doi: 10.1002/cncr.23959

Zikmund-Fisher, B. J., Ubel, P. A., Smith, D. M., Derry, H. A., McClure, J. B., Stark, A., . . . Fagerlin, A. (2008). Communicating side effect risks in a tamoxifen prophylaxis decision aid: the debiasing influence of pictographs. *Patient Educ Couns, 73*(2), 209-214. doi: 10.1016/j.pec.2008.05.010
